# Transcriptional and Post-transcriptional Regulation of Lignin Biosynthesis Pathway Genes in *Populus*

**DOI:** 10.3389/fpls.2020.00652

**Published:** 2020-05-25

**Authors:** Jin Zhang, Gerald A. Tuskan, Timothy J. Tschaplinski, Wellington Muchero, Jin-Gui Chen

**Affiliations:** ^1^Biosciences Division, Oak Ridge National Laboratory, Oak Ridge, TN, United States; ^2^Center for Bioenergy Innovation, Oak Ridge National Laboratory, Oak Ridge, TN, United States

**Keywords:** lignin biosynthesis, plant cell wall, transcriptional regulation, post-transcriptional regulation, transcription factor

## Abstract

Lignin is a heterogeneous polymer of aromatic subunits derived from phenylalanine. It is polymerized in intimate proximity to the polysaccharide components in plant cell walls and provides additional rigidity and compressive strength for plants. Understanding the regulatory mechanisms of lignin biosynthesis is important for genetic modification of the plant cell wall for agricultural and industrial applications. Over the past 10 years the transcriptional regulatory model of lignin biosynthesis has been established in plants. However, the role of post-transcriptional regulation is still largely unknown. Increasing evidence suggests that lignin biosynthesis pathway genes are also regulated by alternative splicing, microRNA, and long non-coding RNA. In this review, we briefly summarize recent progress on the transcriptional regulation, then we focus on reviewing progress on the post-transcriptional regulation of lignin biosynthesis pathway genes in the woody model plant *Populus*.

## Introduction

Lignin is one of the most abundant biopolymers, accounting for ∼30% of the organic carbon in the biosphere. As a principal component of secondary cell walls, lignin provides plants with structural integrity and a response mechanism to environmental stimuli, e.g., pathogen attack. In addition, lignin supports transport of water and solutes through the vascular system. The lignin structure varies between plant species, between cell types within a single plant, and between different parts of the wall of a single cell. The lignin polymer is primarily comprised of three major monomers: *p*-hydroxyphenyl (H), guaiacyl (G), and syringyl (S) monolignols that are synthesized via the phenylpropanoid pathway (Raes et al., 2003). From *Arabidopsis* genome-wide analysis and mutant/transformation studies, at least 14 structural genes have been characterized and shown to be involved in the monolignol biosynthesis pathway (Goujon et al., 2003a).

Although the regulatory mechanism of lignin biosynthesis has been studied in several plant species (Zhong et al., 2006; Zhong and Ye, 2011; Xie et al., 2018b; Zhang et al., 2018a), many aspects of its regulation remain unresolved. Identification of *cis*-acting elements in monolignol biosynthetic genes provides an understanding of the transcriptional regulation of lignin biosynthesis. Promoter analysis and electrophoretic mobility shift assay have revealed that the SNBE (Zhong et al., 2010a) and AC elements (Zhong and Ye, 2011) (corresponding to the NAC and MYB transcription factor-binding motif, respectively) are necessary for coordinated monolignol pathway gene activation. However, a comprehensive understanding of the transcriptional and post-transcriptional regulation of lignin biosynthesis in woody species is still lacking. In this review, we summarize the current understanding of the regulation of lignin biosynthesis pathway genes at the transcriptional level, then focus on the emerging area of post-transcriptional regulation.

## Transcriptional Regulation of Lignin Biosynthesis Pathway Genes

### Structural Genes of Monolignol Biosynthesis

**FIGURE 1:**
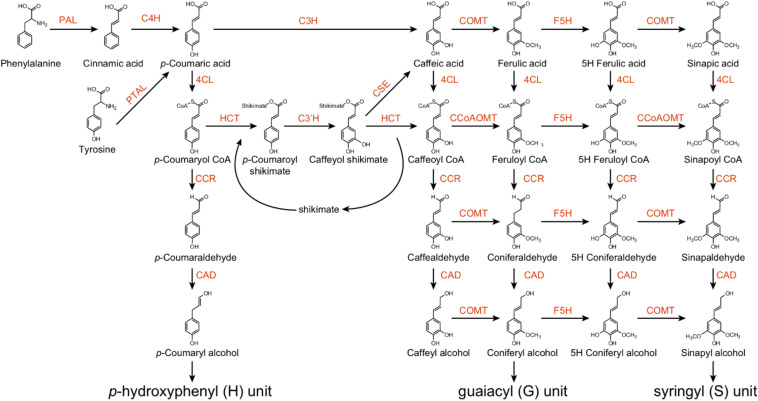
The monolignol biosynthetic pathway in *Populus*. PAL, _L_-phenylalanine ammonia-lyase; PTAL, bifunctional _L_-phenylalanine/_L_-tyrosine ammonia-lyase; C4H, cinnamate 4-hydroxylase; C3H, *p*-coumarate 3-hydroxylase; COMT, caffeate/5-hydroxyferulate 3-O-methyltransferase; F5H, ferulate 5-hydroxylase/coniferaldehyde 5-hydroxylase; 4CL, 4-hydroxycinnamate:CoA ligase; HCT, *p*-hydroxycinnamoyl CoA:shikimate/quinate hydroxycinnamoyltransferase; C3′H, *p*-coumaroyl shikimate/quinate 3′-hydroxylase; CSE, caffeoyl shikimate esterase; CCoAOMT, caffeoyl CoA 3-O-methyltransferase; CCR, cinnamoyl CoA reductase; CAD, cinnamyl alcohol dehydrogenase.

The monolignol biosynthesis pathway has been well studied in several model plant species, such as the model herbaceous species *Arabidopsis* and the model woody species *Populus*. Monolignols are synthesized from phenylalanine via the phenylpropanoid pathway, which includes a series of enzymes controlling alternate linear steps, ultimately providing precursors for numerous secondary metabolites (Fraser and Chapple, 2011). Wang et al. (2018) demonstrated the importance of phenylpropanoid biosynthetic enzymes for lignin biosynthesis in *Populus* using 221 independent transgenic lines derived from 21 lignin biosynthetic genes. These enzymes belong to an assembly of genes and gene families, including phenylalanine ammonia lyase (PAL), cinnamate 4-hydroxylase (C4H), 4-coumarate:CoA ligase (4CL), *p*-coumaroyl-shikimate/quinate 3-hydroxylase (C3H), hydroxycinnamoyl-CoA shikimate/quinate hydroxycinnamoyl transferase (HCT), caffeoyl-CoA *O*-methyltransferase (CCoAOMT), 5-hydroxyconiferyl aldehyde *O*-methyltransferase (AldOMT), coniferyl aldehyde/ferulate 5-hydroxylase (CAld5H/F5H), cinnamoyl-CoA reductase (CCR), cinnamyl alcohol dehydrogenase (CAD), caffeoyl shikimate esterase (CSE), and caffeic acid *O*-methyltransferase (COMT) (Figure 1). PAL, C4H and 4CL play important roles to provide precursors for various downstream metabolites (Figure 1). Down-regulation of PAL, C4H or 4CL can significantly decrease lignin content in both *Arabidopsis* and *Populus* (Rohde et al., 2004; Chen and Dixon, 2007; Vanholme et al., 2008; Wang et al., 2018). Recently, a C3H enzyme is identified as a bifunctional peroxidase that oxidizes both ascorbate and 4-coumarate in the model plants *Brachypodium distachyon* and *Arabidopsis* by directly catalyzing the 3-hydroxylation of 4-coumarate to caffeate in lignin biosynthesis pathway (Barros et al., 2019).

*Populus* is a promising feedstock for biofuels and other value-added products due to its fast growth and high efficiency of biofuel conversion. In addition, abundant public genomics, and transcriptomics resources of *Populus* provide the basis for functional study. Here we focus on *Populus* to explore the transcriptional and post-transcriptional regulation of lignin biosynthetic genes. On the basis of findings reported in literature, we build a conceptual network of the enzymes that control monolignol biosynthesis in *Populus*. As shown in Table 1, the 21 enzymes reported by Wang et al. (2018), and three other enzymes [CSE1, CSE2 (Vanholme et al., 2013) and COMT2 (Marita et al., 2001)], play important roles in monolignol biosynthesis in *Populus* and *Arabidopsis*. We analyzed the expression profiles of the structural genes in monolignol biosynthesis pathway across various tissues and during wood formation in *Populus* based on the *Populus* Gene Expression Atlas database (different tissues of buds, male catkins, female catkins, leaf, root and stem of *P. trichocarpa*, 72 RNA-Seq libraries)^1^ and AspWood database (micro meter-scale profile of *P. tremula* cambial growth and wood formation, 137 RNA-Seq libraries) (Sundell et al., 2017).

**TABLE 1:** Monolignol biosynthetic genes in *Populus*.

**Gene ID**	**Gene family**	**Enzyme**	**Substrate**
Potri.006G126800	*PAL*	PAL1	Phe
Potri.008G038200	*PAL*	PAL2	Phe
Potri.016G091100	*PAL*	PAL3	Phe
Potri.010G224100	*PAL*	PAL4	Phe
Potri.010G224200	*PAL*	PAL5	Phe
Potri.013G157900	*C4H*	C4H1	Cinnamic acid
Potri.019G130700	*C4H*	C4H2	Cinnamic acid
Potri.001G036900	*4CL*	4CL3	4-coumaric acid, caffeic acid, ferulic acid, 5-hydroxyferulic acid
Potri.003G188500	*4CL*	4CL5	Caffeic acid, 4-coumaric acid, ferulic acid, 5-hydroxyferulic acid, sinapic acid
Potri.006G033300	*C3H*	C3H3	4-coumaroyl shikimic acid, 4-coumaric acid
Potri.003G183900	*HCT*	HCT1	4-coumaroyl-CoA, 4-coumaroyl shikimic acid, caffeoyl-CoA, caffeoyl shikimic acid
Potri.001G042900	*HCT*	HCT6	4-coumaroyl-CoA, 4-coumaroyl shikimic acid, caffeoyl-CoA, caffeoyl shikimic acid
Potri.009G099800	*CCoAOMT*	CCoAOMT1	Caffeoyl-CoA
Potri.001G304800	*CCoAOMT*	CCoAOMT2	Caffeoyl-CoA
Potri.008G136600	*CCoAOMT*	CCoAOMT3	Caffeoyl-CoA
Potri.015G119600	*AldOMT*	AldOMT2	Caffealdehyde, 5-hydroxyconiferaldehyde, caffeyl alcohol, 5-hydroxyconiferyl alcohol, 5-hydroxyferulic acid, caffeic acid
Potri.005G117500	*CAld5H/F5H*	CAld5H1, F5H1	Coniferyl alcohol, coniferaldehyde, ferulic acid
Potri.007G016400	*CAld5H/F5H*	CAld5H2, F5H2	Coniferyl alcohol, coniferaldehyde, ferulic acid
Potri.003G181400	*CCR*	CCR2	Feruloyl-CoA, 4c-oumaroyl-CoA, caffeoyl-CoA
Potri.009G095800	*CAD*	CAD1	Coniferaldehyde, 4-coumaraldehyde, sinapaldehyde
Potri.016G078300	*CAD*	CAD2	Sinapaldehyde, coniferaldehyde
Potri.003G059200	*CSE*	CSE1	Caffeoyl shikimate
Potri.001G175000	*CSE*	CSE2	Caffeoyl shikimate
Potri.012G006400	*COMT*	COMT2	Caffeic acid, caffeoyl-CoA, caffeoyl aldehyde, caffeoyl alcohol

**FIGURE 2:**
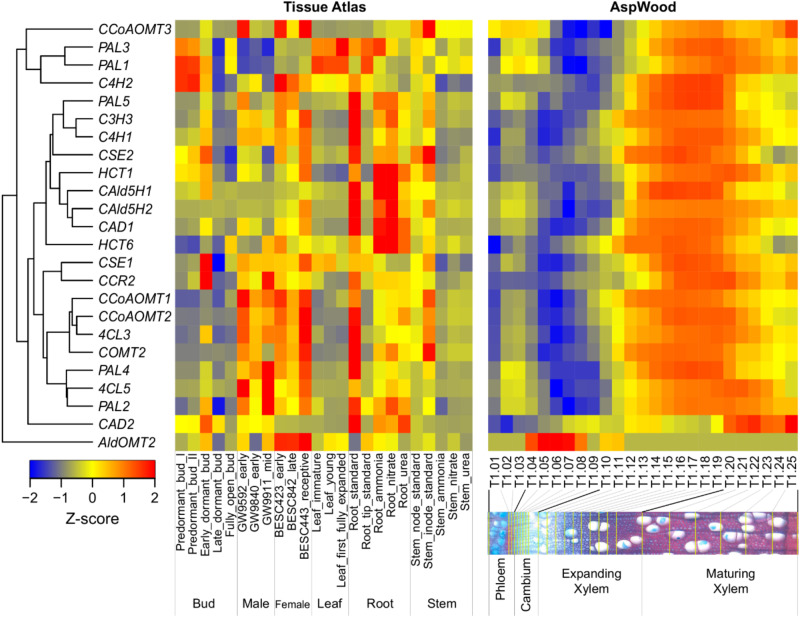
Hierarchical clustering of the expression profiles of monolignol biosynthetic genes across various tissues (left) and during wood formation (right) in *Populus*. The expression data of different tissues and wood formation were obtained from *Populus* Gene Expression Atlas (https://phytozome.jgi.doe.gov/phytomine/aspect.do?name=Expression) and AspWood (http://aspwood.popgenie.org/aspwood-v3.0/), respectively. The tissue atlas dataset includes tissues collected from buds, male catkins, female catkins, leaf, root and stem. The AspWood dataset includes samples collected from phloem, cambium, expanding xylem and maturing xylem. Gene expression was normalized by Z-score. Red and blue represent high and low expression, respectively.

Broad expression evidence from key enzymes in the lignin biosynthetic pathway provides a hypothetical foundation for their functions in various tissues. For example, Kim et al. (2019) performed a series of wood-forming tissue-specific transcriptome analyses from a hybrid poplar and identified critical pathway genes for secondary wall biosynthesis in mature developing xylem. Wood formation is a process of plant secondary growth, which originates from the cambium meristem cells, eventually forming a tree’s main stem or truck. Most of the genes involved in this process are highly expressed in the developing xylem. In contrast, *CAD2* and *AldOMT2* are highly expressed in maturing xylem and cambium, respectively (Figure 2). In a promoter-GUS histochemistry analysis, the GUS driven by promoter of *Eucalyptus gunnii CAD2* is expressed in all lignifying cells including vessel elements, xylem fibers and paratracheal parenchyma cells of the xylem tissues in the transgenic *Arabidopsis* floral stem and root (Baghdady et al., 2006). The expression pattern and function of *AldOMT2* homologs remains unclear.

### Transcription Factors Involved in the Lignin Biosynthesis Pathway

A hierarchical transcriptional regulatory network for lignin biosynthetic genes has been established over the past 10 years (Zhao et al., 2010; Zhong and Ye, 2011; Lin et al., 2017; Zhang et al., 2018a; Chen et al., 2019). This network involves members of several transcription factor (TF) families including MYBs and NACs. A recent study identified a novel TF (i.e., PtrEPSP-TF) encoding a homolog of 5-enolpyruvylshikimate 3-phosphate (EPSP) synthase in the shikimate pathway, which possesses a helix-turn-helix motif in the N terminus and can function as a transcriptional repressor to regulate gene expression in the phenylpropanoid pathway in *Populus* (Xie et al., 2018a). Correspondingly, the expression of lignin-related TFs is affected by several other genes. For example, overexpression of a serine hydroxymethyltransferase (*PtSHMT2*) decreases the lignin content in transgenic poplar (Zhang et al., 2019a). Overexpression of a prefoldin chaperonin β subunit gene *PdPFD2.2* increases lignin S/G ration in poplar (Zhang et al., 2019b). This suggests that the molecular regulation of lignin biosynthesis is not unidirectional and is more complex than that was previously reported.

**FIGURE 3:**
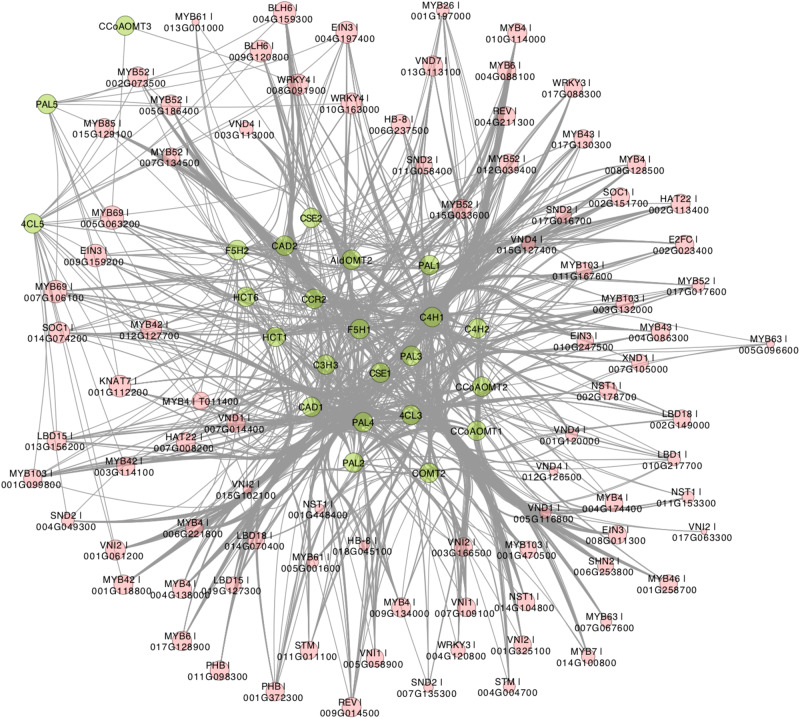
Regulatory network generated by triple-gene mutual interaction (TGMI) algorithm for the *Populus* lignin biosynthesis pathway using the RNA-Seq data from *Populus* Gene Expression Atlas and AspWood datasets. Green nodes represent monolignol biosynthetic genes. Red nodes are transcription factors (TF) and node size represent frequency of TF. Edges represent regulatory relationships from TGMI algorithm.

**FIGURE 4:**
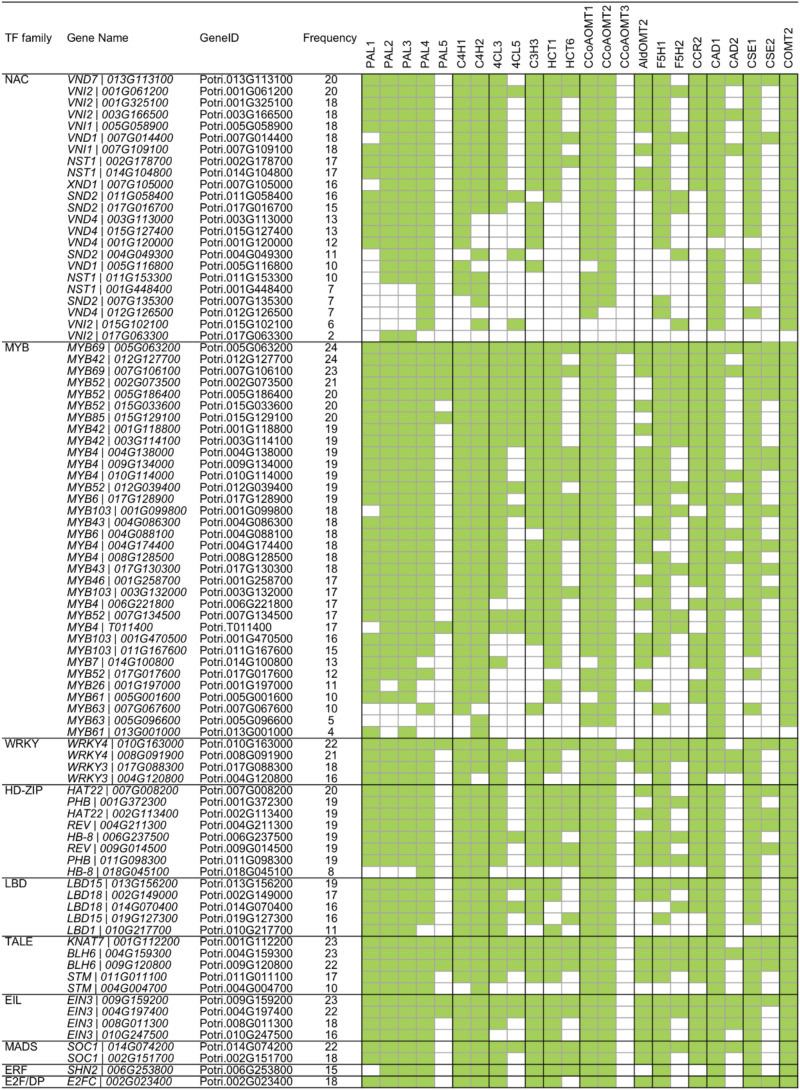
Regulatory relationship of transcription factor (TF) and monolignol biosynthetic genes generated by triple-gene mutual interaction (TGMI) algorithm. Green blocks represent statistically significant interactions.

Recently, Gunasekara et al. (2018) developed a novel algorithm called triple-gene mutual interaction (TGMI) for identifying the pathway regulators using high-throughput gene expression data, which calculates the mutual interaction measure for each triple gene grouping (two pathway genes and one TF) and then examines its statistical significance using bootstrap. Implementing this algorithm, Gunasekara et al. (2018) analyzed pathway regulators of lignin biosynthesis using a compendium dataset that comprised 128 microarray samples from *Arabidopsis* stem tissues under short-day conditions. In this review, we also applied the TGMI algorithm to identify regulators of lignin biosynthesis in *Populus* based on the tissue-specific *Populus* Gene Expression Atlas and AspWood datasets (209 RNA-Seq samples in total). As anticipated, a series of known lignin biosynthesis-related TFs (87 TFs from 10 families), such as members in NAC and MYB families, were correlated with the lignin biosynthetic genes (Figures 3, 4). In addition, we identified several novel TFs that were highly correlated with the monolignol biosynthetic genes, expanding our view of the transcriptional regulatory network affecting lignin biosynthesis. Individual classes of these TFs are presented in Figures 3, 4.

### Transcriptional Regulation of Lignin Biosynthetic Genes

#### PAL

To further understand the transcriptional regulation between TFs and lignin biosynthetic genes, we generated a heatmap to reveal the correlation between lignin biosynthetic genes and known lignin-related TFs (Figure 4). PAL genes showed strong correlation with MYB TFs. During secondary cell wall formation, *MYB46* and *MYB83* and their orthologs in several plant species, including *Arabidopsis*, *Populus*, and *Eucalyptus*, have been identified as the direct targets of SNDs (SECONDARY WALL-ASSOCIATED NAC DOMAIN PROTEINS) and VNDs (VASCULAR-RELATED NAC DOMAINS) and function as the second-layer master switches (McCarthy et al., 2010; Zhong and Ye, 2011; Kim et al., 2013; Ko et al., 2014; Zhang et al., 2018a). Overexpression of *MYB46* and *MYB83* caused ectopic deposition of secondary cell walls through activation of the lignin, cellulose and xylan biosynthetic genes (Zhong and Ye, 2011). Electrophoretic mobility shift assay and chromatin immunoprecipitation analysis showed that MYB46 directly binds to the promoters of PAL (Kim et al., 2014). Similarly, their orthologs in *Populus*, *PtrMYB3* (Potri.001G267300) and *PtrMYB20* (Potri.009G061500), activate the biosynthesis pathways of lignin, cellulose and xylan in both *Arabidopsis* and *Populus*, including *PAL* genes (McCarthy et al., 2010). In *Populus tomentosa, PtoMYB216* (GenBank: JQ801749, ortholog of Potri.013G001000), a homolog of *Arabidopsis AtMYB61* and *AtMYB85*, was specifically expressed during secondary wall formation in wood. The expression of *PAL4* was induced in the transgenic plants overexpressing *PtoMYB216* (Tian et al., 2013). *PtoMYB156* (GenBank: KT990214, ortholog of Potri.009G134000) is a homolog of *AtMYB4*, which functions as phenylpropanoid/lignin biosynthesis repressor. Overexpression of *PtoMYB156* in poplar also resulted in downregulation of *PtoPAL1* (Yang et al., 2017). Four additional MYB TFs (MYB20, MYB42, MYB43 and MYB85) were recently reported as transcriptional regulators that directly activate lignin biosynthetic genes during secondary wall formation in *Arabidopsis*. Quadruple mutant *myb20/42/43/85* plants exhibited reduced transcript levels of *PAL* (Geng et al., 2020). From these results, MYB TFs appear to be regulated by a series of master switches during secondary cell wall biosynthesis. The transcriptional regulation of *PAL* is likely regulated by a hierarchical or more complex pattern, in addition to the direct regulation by these MYB TFs.

#### C4H

As shown in Figure 4, *C4H1* was correlated with the TGMI-based expression of 32 *MYB* TFs. Recently, a transcriptional regulatory network (TRN) of wood formation based on a *P. trichocarpa* wood-forming cell system with quantitative transcriptomics and chromatin binding assays was constructed (Chen et al., 2019). In the TRN, *PtrC4H1* was regulated by *PtrWBLH2* (a wood Bel-like homeodomain protein), which is a direct target of *PtrMYB021* and *PtrMYB074*. Comparably, in *P. tomentosa*, *C4H2* is directly activated by *PtoMYB216* through AC elements (Tian et al., 2013). In addition, the expression of *C4H* was repressed by *MYB* transcriptional repressors. In *Arabidopsis*, *AtMYB4* downregulates the expression of *C4H* (Jin et al., 2000). Ectopic expression of *E. gunnii EgMYB1* in *Populus* repressed the expression of *PtaC4H2* in wood tissue (Legay et al., 2010). Moreover, *Arabidopsis WRKY12* is a transcriptional repressor that can directly bind to the promoter of *NST2*, a master regulator of lignin biosynthesis. Loss-of-function mutants of *WRKY12* in *Arabidopsis*, and its ortholog in *Medicago*, result in ectopic deposition of lignin, xylan, and cellulose in pith cells (Wang et al., 2010). Its homolog in *Populus*, *PtrWKRY19* (Potri.014G050000), is highly expressed in stems, especially in pith. Finally, *PtrWRKY19* can repress the expression of *PtoC4H2* through W-box elements (Yang et al., 2016).

#### 4CL

4CL is the third step in the phenylpropanoid pathway and it is important for not only monolignol biosynthesis but also the generation of other secondary metabolites (Tsai et al., 2006). Based on the regulatory network, the two *4CL* genes (*4CL3* and *4CL5*) were correlated with multiple NAC and MYB TFs (Figure 4). In *Populus*, the expression of *4CL5* was upregulated in transgenic plants overexpressing *PtrMYB152* (GenBank: XM_002302907, ortholog of Potri.017G130300), a homolog of *AtMYB58/63/85* (Li et al., 2014). Similarly, *4CL5* could be activated by another MYB member *PtoMYB216* (Tian et al., 2013). The promoters of *4CL* genes include AC elements that provide binding sites for secondary cell-wall-related MYB genes. In several plant species, NAC TFs have been reported to regulate the expression of *4CL* genes. In support of these observations, *EjNAC1* had trans-activation activities on promoter of *Ej4CL1* (Xu et al., 2015) and the expression of *4CL* was repressed in *Medicago nst* mutant (Zhao et al., 2010). However, whether *4CL* genes are direct targets of NAC TFs in *Populus* remains unknown.

#### C3H

The regulatory network pattern in Figure 4 reveals that *C3H* has a similar pattern to the *4CL* genes, indicating the transcriptional regulation of *C3H* might be similar with *4CL* genes. As expected, the expression of *C3H3* was also activated by *PtoMYB216* and *PtrMYB152* (Tian et al., 2013; Li et al., 2014). Still, studies of other species revealed that C3H could be regulated by other TF families. Switchgrass *PvMYB4* is a transcriptional repressor and binds to the AC elements. The expression of *C3H* was activated by overexpressing *PvMYB4* in transgenic tobacco and switchgrass (Shen et al., 2012). In *Medicago nst* mutant, the expression of *C3H* was repressed due to loss-of-function of *NST* (Zhao et al., 2010). In addition, the expression of *C3H* was induced by overexpressing *GbERF1-like*, a *Gossypium barbadense* ethylene response-related factor, in transgenic cotton and *Arabidopsis* (Guo et al., 2016). The AC elements provide the binding sites for the direct TF regulation.

#### HCT

HCT is involved in the production of methoxylated monolignols that are precursors to G- and S-unit lignin. HCT-downregulated plants are strikingly enriched in H lignin units, a minor component of lignin (Wagner et al., 2007). In *P. trichocarpa*, *HCT1* and *HCT6* display xylem-specific expression, which is regulated by *PtrWBLH2* and *PtrWBLH1*, respectively (Chen et al., 2019). A recent study using genome-wide association studies (GWAS) and expression quantitative trait loci (eQTL)/expression quantitative trait nucleotide (eQTN) studies identified a defense-related *HCT2* that was regulated by *WRKY* TFs (Zhang et al., 2018b), implying that other TF families might be also involved in the transcriptional regulation of *HCT* gene family under alternate developmental circumstances. Heterologous expressing *SbbHLH1*, a *Sorghum bicolor* basic helix-loop-helix gene, reduced the lignin content through repress the expression of *HCT* in transgenic *Arabidopsis* (Yan et al., 2013).

#### CCoAOMT

As shown in Figure 4, three *CCoAOMT* genes were highly positively correlated with seven TFs in NAC family. It has been reported that NAC TFs function as master regulators in the lignin biosynthesis pathway. The SECONDARY WALL NACs (SWNs) consists of two types NACs: SECONDARY WALL-ASSOCIATED NAC DOMAIN PROTEIN (SND)/NAC SECONDARY WALL THICKENING PROMOTING FACTOR (NST) and VASCULAR-RELATED NAC DOMAINS (VNDs) (Zhang et al., 2018a). In *Arabidopsis*, ectopic overexpression of *SND1* significantly induced the expression of CCoAOMT (Zhong et al., 2006). In *Populus*, six SND1 homologs, named *PtrWND1-6* (WOOD ASSOCIATED NAC DOMAIN), are highly expressed in the developing xylem. Overexpression of *PtrWND2B* and *PtrWND6B* in *Arabidopsis* causes ectopic deposition of secondary cell wall through activation of the lignin, cellulose and xylan biosynthetic genes (Zhong et al., 2010b). In *Populus*, the transcript of *CCoAOMT1* was induced by overexpressing WND3A (Yang et al., 2019). Zhou et al. (2014) demonstrated that the promoter of *CCoAOMT1* is directly activated by *Arabidopsis* VND1-5. Similar results were also found in *Arabidopsis* transgenic lines expressing PtrWND6B. A transactivation assay indicates *CCoAOMT* is direct target of PtrWND6B (Zhong et al., 2010b). In addition, MYB TFs were also involved in transcriptional regulation of *CCoAOMT*. As direct target of PtrWND2, PtrMYB3 and PtrMYB20 (homologous of *Arabidopsis* MYB46/83) were able to activate the promoters of *PtrCCoAOMT1* through *Arabidopsis* protoplast transactivation analysis (McCarthy et al., 2010).

#### CAld5H/F5H

F5H is a cytochrome P450 (CYP)-dependent monooxygenase, it is specifically required for S-unit lignin biosynthesis and diverts G-unit into the S-unit pathway (Humphreys et al., 1999). Using *P. trichocarpa* wood-forming cell system, three TFs (*PtrMYB090*, *PtrMYB161* and *PtrWBLH2*) were identified as upstream regulator of *F5H* genes in *Populus* (Chen et al., 2019). In *Medicago*, the expression of F5H is directly regulated by the secondary cell wall master switch NST1/SND1 (Zhao et al., 2010). In addition, *MYB103* is required for the expression of *F5H* and S-lignin biosynthesis in *Arabidopsis*. The S-lignin content, as well as transcript level of *F5H*, are strongly decreased in the *myb103* mutants, whereas the G-lignin content was concomitantly increased (Öhman et al., 2013).

#### CCR and CAD

CCR and CAD catalyze the final steps of monolignol biosynthesis (Figure 1). In many species, CCR and CAD exhibit similar expression patterns in vascular tissues. The expression of *PtrCAD1* was repressed by *PtrMYB174* in *Populus* (Chen et al., 2019). Other studies indicated that *CCR2* and *CAD* were activated by *PtoMYB216* and *PtrMYB152* (Tian et al., 2013; Li et al., 2014). Using promoter deletion analysis, Rahantamalala et al. (2010) identified an 80-bp region and a 50-bp region in the promoters of *E. gunnii EgCAD2* and *EgCCR* that contains MYB elements, respectively. In addition, heterologous expressing *Vitis vinifera VvWRKY2* activate the expression of *CCR* and *CAD* in transgenic tobacco (Guillaumie et al., 2010).

#### CSE

CSE is a recently identified novel enzymatic step in the lignin biosynthetic pathway (Vanholme et al., 2013). Similar to other MYB46/83 regulated genes, *CSE* has M46RE motifs in the promoter region, and its expression is induced by *MYB46* (Kim et al., 2014). In *Populus*, it is directly regulated by *PtrWBLH1*, a downstream regulator of *PtrMYB021* (homolog of *Arabidopsis MYB46*) (Chen et al., 2019). In addition, the regulatory network indicated that *CSE1* is negatively correlated with a *WRKY* TF in *Populus* (Figure 4), but whether *WRKY* directly regulates *CSE* needs to be confirmed.

#### COMT

COMT is critical for the S-unit lignin biosynthesis (Goujon et al., 2003b). In *Arabidopsis*, *COMT* is directly regulated by a lignin-specific MYB *AtMYB58* through binding to the AC elements (Zhou et al., 2009). A similar regulatory pattern is also observed in *Populus*. That is, *COMT2* is activated by *PtoMYB170*, *PtrMYB090* and *PtrMYB152*, but not *PtoMYB216* (Tian et al., 2013; Li et al., 2014; Xu et al., 2017; Chen et al., 2019). In addition, the promoter of *Arabidopsis COMT* could be bound by BP, a knotted1-like homeobox (KNOX) gene (Mele et al., 2003). The TGMI analysis indicated that *COMT2* is highly associated with TFs in HD-ZIP and LBD families, in addition to NAC and MYB TFs (Figure 4). However, experimental evidence will be required to verify this regulatory relationship.

## Post-Transcriptional Regulation of Lignin Biosynthesis Pathway Genes

Post-transcriptional regulation of lignin biosynthesis pathway genes plays important roles in molecular regulation at the RNA level, including controlling alternative splicing, RNA capping, poly-A tail addition, and mRNA stability (Sullivan and Green, 1993). To date, studies of the post-transcriptional regulation of lignin pathway have been focused on transcriptional regulatory genes. In this section, we summarize recent progress on the post-transcriptional regulation of regulatory genes in lignin pathway.

### Alternative Splicing

Alternative splicing, as a post-transcriptional regulation mechanism, allows organisms to increase their proteomic diversity and regulate gene expression. It has been reported that alternative splicing of key regulators and enzymes play a critical role in the lignin biosynthesis pathway. A previous study analyzed the transcriptome of 20 *P. trichocarpa* individuals and found that ∼40% xylem genes are alternatively spliced, which include cell wall-related genes C2H2 TF and glycosyl transferases (Bao et al., 2013). Xu et al. (2014) compared the inter-species conservation of alternative splicing events in the developing xylem of *Populus* and *Eucalyptus* and found that ∼28% of alternative splicing genes were putative orthologs in these two species. Alternative splicing can also affect the expression of downstream genes. For example, retention of intron 2 of *Populus PtrWND1B/PtrSND1*, by alternative splicing, resulted in loss of DNA binding and transactivation activities (Li et al., 2012). This alternative splicing event appears to regulate secondary cell wall thickening and the expression of the lignin-related gene *4CL1*. Similar alternative splicing was also observed in its orthologs in *Eucalyptus*, but not in *Arabidopsis* (Zhao et al., 2014). In addition, other members in the VND- and SND-type NAC family are regulated by alternative splicing. For example, retained introns of *PtrSND1-A2* and *PtrVND6-C1* play reciprocal cross-regulation of the two families during wood formation (Lin et al., 2017).

### microRNA

microRNAs (miRNAs) are a class of small non-coding RNAs with a 21-23 ribonucleotide RNA sequence that play central roles in gene expression regulation through directing mRNA cleavage or translational inhibition. Several miRNAs, such as miRNA397, miRNA408, miRNA857, and miRNA528, have been reported to target laccase (LAC) genes, encoding a class of blue copper oxidase proteins involved in lignin polymerization (Sunkar and Zhu, 2004; Lu et al., 2013). In *Populus*, the expression of 17 *PtrLACs* are down-regulated and lignin content is decreased by overexpression of *Ptr-miRNA397a* (Lu et al., 2013). *Arabidopsis LAC4* controls both lignin biosynthesis and seed yield, and its expression is controlled by *miRNA397* member *At-miRNA397b*. Overexpression of *At-miRNA397b* reduced lignin deposition through repression of the biosynthesis of both S- and G-lignin subunits (Wang et al., 2014). In addition, overexpression a wounding-responsive *miRNA828* can enhance lignin deposition and H_2_O_2_ accumulation through repressed expression of *IbMYB* and *IbTLD* in sweet potato (Lin et al., 2012). *Acacia mangium* miRNA166 is differentially expressed between phloem and xylem, where it targets HD-ZIP III type TFs to regulate the expression of *C4H*, *CAD*, and *CCoAOMT* (Ong and Wickneswari, 2012). In maize, *Zm-miRNA528*, induced by excess nitrogen and repressed by nitrogen deficiency, targets *LAC3* and *LAC5* and regulates the biosynthesis of S-, G-, and H-subunits (Sun et al., 2018). Finally, in *Arabidopsis*, *miRNA858a* directly regulates the expression of several *MYBs* during flavonoid biosynthesis. Overexpression of *miRNA858a* results in ectopic deposition of lignin in transgenic plants (Sharma et al., 2016). Collectively, these results indicate that miRNAs play important regulatory roles during multiple levels of lignin biosynthesis.

### Long Non-coding RNA

Long non-coding RNAs (lncRNAs) refer to transcripts that lack coding potential and are greater than 200 nucleotides (Kapranov et al., 2007). Chen et al. (2015) performed a genome-wide identification of lncRNA in tension wood, opposite wood and normal wood xylem of *P. tomentosa* and identified 16 genes targeted by lncRNAs that are involved in wood formation processes, including lignin biosynthesis (Chen et al., 2015). In a similar study, the interaction of NEEDED FOR RDR2-INDEPENDENT DNA METHYLATION (NERD) and its regulatory lncRNA NERDL, which is partially located within the promoter region of NERD, is involved in the wood formation processes in *Populus* (Shi et al., 2017). In cotton, Dt subgenome-specific lncRNAs are enriched in lignin catabolic processes. Wang et al. (2015) suggests that these lncRNAs may regulate lignin biosynthesis by regulating the expression of *LAC4* (Wang et al., 2015). Although these studies imply the potential roles of lncRNAs in lignin biosynthesis, the underlying regulatory mechanism remain unverified.

## Concluding Remarks

In this review, we provide a comprehensive summary of the current knowledge of the transcriptional regulation of lignin biosynthetic genes and post-transcriptional regulation of regulatory genes in lignin biosynthesis in *Populus*. Lignin content has been reported as important factor in biomass recalcitrance for bioethanol conversion and production. Although many genes that play a regulatory role in the lignin biosynthesis pathway were captured in TGMI analysis, some previously reported lignin pathway regulators were missing, possibly due to limited data in our analysis. To overcome this issue and to capture other regulatory genes, multiple datasets, pooled from various tissues types during specific rapid developmental processes, should be investigated. In addition, GWAS and eQTL/eQTN analyses may provide further supportive lucidity in discovering novel regulators and regulatory mechanisms in lignin biosynthesis. Revealing the transcriptional and post-transcriptional regulatory mechanisms in lignin biosynthesis will help clarify the parameters of the lignin biosynthesis, ultimately improving the application of lignocellulose in biofuels and bioenergy. Understanding the increasingly complex lignin regulatory network will provide an important theoretical basis for basic plant biology and utilization of plant biomass.

## Authors’ Note

This manuscript has been authored by UT-Battelle, LLC under Contract No. DE-AC05-00OR22725 with the U.S. Department of Energy. The United States Government retains and the publisher, by accepting the article for publication, acknowledges that the United States Government retains a non-exclusive, paid-up, irrevocable, worldwide license to publish or reproduce the published form of this manuscript, or allow others to do so, for United States Government purposes. The Department of Energy will provide public access to these results of federally sponsored research in accordance with the DOE Public Access Plan (http://energy.gov/downloads/doe-public-access-plan).

## Author Contributions

JZ collected and synthesized the data from literature and wrote the manuscript. GT, TT, WM, and J-GC revised the manuscript.

## Conflict of Interest

The authors declare that the research was conducted in the absence of any commercial or financial relationships that could be construed as a potential conflict of interest.

## Footnotes

1 https://phytozome.jgi.doe.gov/phytomine/aspect.do?name=Expression
